# Supplementary material for: The Systems Biology Research Tool: evolvable open-source software
Source: BMC Syst Biol. 2008 Jun 29;2:55. doi: 10.1186/1752-0509-2-55 (PMC2446383; doi:10.1186/1752-0509-2-55)
Supplement: Additional file 1 — SBRT Archive. An archive of the current version of the Systems Biology Research Tool. [file 1752-0509-2-55-S1.zip › sbrt-1.4.0/doc/developers_guide/api/sbrt/kernel/math/optimization/OptimizationSense.html]

OptimizationSense


|  |  |  |
| --- | --- | --- |
| **Overview** \| **Package** \| **Class** \| **Use** \| **Tree** \| **Deprecated** \| **Index** \| **Help** | |  |
| **PREV CLASS**   **NEXT CLASS** | **FRAMES**    **NO FRAMES**     **All Classes** |  |
| SUMMARY: NESTED \| ENUM CONSTANTS \| FIELD \| METHOD | DETAIL: ENUM CONSTANTS \| FIELD \| METHOD |  |


---


## sbrt.kernel.math.optimization Enum OptimizationSense

```
java.lang.Object
  java.lang.Enum<OptimizationSense>
      sbrt.kernel.math.optimization.OptimizationSense
```

**All Implemented Interfaces:**: java.io.Serializable, java.lang.Comparable<OptimizationSense>

---

``` public enum OptimizationSense extends java.lang.Enum<OptimizationSense> ```

This enum type is used to represent the sense, or direction,
of an optimization problem.

**Author:**
:   This enum type was written and documented by
    Jeremiah Wright while in the Wagner lab.

---

**Enum Constant Summary**

`MAX`
  
          Used to indicate a maximization problem.

`MIN`
  
          Used to indicate a minimization problem.


| **Method Summary** | |
| --- | --- |
| `java.lang.String` | `toString()`             Returns an appropriate string representation of this optimization sense. |
| `static OptimizationSense` | `valueOf(java.lang.String name)`             Returns the enum constant of this type with the specified name. |
| `static OptimizationSense[]` | `values()`             Returns an array containing the constants of this enum type, in the order they're declared. |

**Methods inherited from class java.lang.Enum**

`clone, compareTo, equals, getDeclaringClass, hashCode, name, ordinal, valueOf`

**Methods inherited from class java.lang.Object**

`finalize, getClass, notify, notifyAll, wait, wait, wait`

**Enum Constant Detail**

### MAX

```
public static final OptimizationSense MAX
```

:   Used to indicate a maximization problem.

---


### MIN

```
public static final OptimizationSense MIN
```

:   Used to indicate a minimization problem.


**Method Detail**

### values

```
public static final OptimizationSense[] values()
```

:   Returns an array containing the constants of this enum type, in
    the order they're declared. This method may be used to iterate
    over the constants as follows:

    ```
    for(OptimizationSense c : OptimizationSense.values())
            System.out.println(c);
    ```

    :   **Returns:**: an array containing the constants of this enum type, in the order they're declared

---


### valueOf

```
public static OptimizationSense valueOf(java.lang.String name)
```

:   Returns the enum constant of this type with the specified name.
    The string must match *exactly* an identifier used to declare an
    enum constant in this type. (Extraneous whitespace characters are
    not permitted.)

    :   **Parameters:**: `name` - the name of the enum constant to be returned. **Returns:**: the enum constant with the specified name **Throws:**: `java.lang.IllegalArgumentException` - if this enum type has no constant with the specified name

---


### toString

```
public java.lang.String toString()
```

:   Returns an appropriate string representation
    of this optimization sense.

    :   **Overrides:**: `toString` in class `java.lang.Enum<OptimizationSense>`
    :   **Returns:**: an appropriate string representation of this optimization sense.


---


|  |  |  |
| --- | --- | --- |
| **Overview** \| **Package** \| **Class** \| **Use** \| **Tree** \| **Deprecated** \| **Index** \| **Help** | |  |
| **PREV CLASS**   **NEXT CLASS** | **FRAMES**    **NO FRAMES**     **All Classes** |  |
| SUMMARY: NESTED \| ENUM CONSTANTS \| FIELD \| METHOD | DETAIL: ENUM CONSTANTS \| FIELD \| METHOD |  |


---
